# Editorial

**Published:** 1856-06

**Authors:** 


### EDITORIAL.

We devote a large part of the present number of the Journal to the proceedings of the recent Annual Meeting of the American Medical Association, having no doubt but all our subscribers will be glad to have the whole together. It will be seen, from the number and character of the reports and papers referred to the Committee on Publication, that the next volume of the Transactions will be large, and filled with matter of permanent interest and value. If any of our subscribers wish to secure a copy, when published, they can do so by remitting *three* dollars to the Treasurer, Dr. C. Wistar, of Philadelphia. The number of members in attendance on the meeting in Detroit was quite as large as was expected, and all the business was transacted with harmony and good feeling. The usual social entertainments, in the evenings, were pleasant seasons, contributing much to the aggregated enjoyment of the Association. The steamboat excursion on the St. Clair River was the only thing that struck us as ill-timed or out of place. It was so arranged as to use up the whole afternoon of Wednesday, thereby taking out one-half of the best working day of the Annual Session, and compelling those who had sacrificed much for the sake of attending the *necessary business* of the Association, and anxious to return home at the earliest moment, either to sit idle in their hotels, or participate in pleasures for which they had neither time nor inclination. At all previous Annual Meetings, excursions, and the like, had uniformly been so arranged as not to interfere with any of the hours properly belonging to the business of the Association, and we hope the same rule will be followed hereafter.

